# Editorial: Unravelling the complex and multifaceted role of the cerebellum in health and disease

**DOI:** 10.3389/fnsys.2023.1155939

**Published:** 2023-03-23

**Authors:** Paul James Mathews, Anne-Lise Paradis, Marija Cvetanovic, Erik Sean Carlson, Krystal Lynn Parker

**Affiliations:** ^1^Department of Neurology, University of California, Los Angeles School of Medicine, Los Angeles, Los Angeles, CA, United States; ^2^Department of Neurology and Institute for Neurotherapeutics, Lundquist Institute for Biomedical Innovation, Torrance, CA, United States; ^3^Neurosciences Paris-Seine, Institut de Biologie Paris-Seine (IBPS), Sorbonne Université, CNRS, Inserm, Paris, France; ^4^Department of Neuroscience, Institute for Translational Neuroscience, University of Minnesota Twin Cities, St. Paul, MN, United States; ^5^Department of Psychiatry and Behavioral Sciences, University of Washington, Seattle, WA, United States; ^6^Geriatric Research, Education, and Clinical Center at the Veteran's Affairs Puget Sound Medical Center, Seattle, WA, United States; ^7^Department of Psychiatry and the Iowa Neuroscience Institute, The University of Iowa, Iowa City, IA, United States

**Keywords:** cerebellum, cerebellum-forebrain connectivity, movement disorders, developmental anomalies, computational modeling, cognition, emotion, pain

The cerebellum is a fascinating brain area to research. It contains most of the brain's neurons, is composed of a highly structured and stereotypical arrangement of cells, has a similar cortical area as the cerebrum, is highly interconnected with the forebrain and brainstem, and has expanded in size over the course of evolution to sustain increased mental and physical capacity. For over 100 years, specific dysfunction in or damage to the cerebellum has been known to cause dramatic deficits in motor coordination. Now, with novel behavioral assessment and advanced technological tools, including computational modeling, functional neuroimaging, advanced tract tracing methods, neuromodulatory tools, and the ability to directly modify gene expression, mounting evidence has dramatically broadened the fields perspective of the cerebellum's role in healthy behavior, implicating it in regulating cognition, mood, reward, decision making, pain, and addiction.

The purpose of this Editorial Research Topic is to collect new and important research focused on how the interconnectivity of the cerebellum with the rest of the brain shapes a wide breadth of functions underlying typical behavior and may explain disease related dysfunction.

Major advancements in our understanding of the cerebellum's role in behavior derive from advances in tract tracing, neural circuit studies, computational modeling approaches, and functional neuroimaging. In the review by McAfee et al., the authors provide a new perspective on cerebellum-forebrain interaction in which the cerebellum coordinates neuronal communication between multiple cerebral cortical regions in a task dependent manner. The authors review studies supporting the cerebellum's role in coordinating cerebral/forebrain activity by influencing cerebral oscillations. The importance of cerebellum-forebrain interactions is further highlighted by Jung et al., where they report using transneuronal tracing techniques and *ex vivo* optophysiology to demonstrate an indirect cerebellar input to the amygdala. This circuit provides a pathway for the cerebellum to impact emotion and potentially pain as discussed in the article by Wang et al. In their review, they detail several downstream potential targets of the cerebellum, including the amygdala, where the cerebellum could disrupt normal circuit function to induce perceptions of pain in migraine headaches. Woodward et al. provide another example of what can happen when cerebellum-forebrain communication becomes dysregulated, detailing in their review how changes in the neural signaling dynamics of the cerebello-thalamo-cortical network in a harmaline-induced mouse model may be a key feature of the motor disease, essential tremor. These articles highlight the diverse array of known and still to be discovered areas of the brain in which the cerebellum has the potential to normally or abnormally influence neural activity.

Behavioral and computational/theoretical modeling studies provide complementary insight on how cerebro-cerebellar networks are organized and what type of signal processing they sustain. The review article by Laurens presents a novel theoretical framework built on prior experimental findings. He posits that the nodulus and uvula (NU) of the cerebellum contributes to the perception of head rotation, positional tilt with respect to gravity, translational motion, and helps distinguish self-generated from externally induced involuntary head movements *via* an internal model that predicts otolith activation to provide downstream areas with sensory prediction errors. This computational approach emphasizes the close interaction between motor control loops and sensation, explaining the clinical consequences of NU lesions, such as increased postural disturbance under conditions where motor feedback is altered. This is in line with what is reported by Guinamard et al. in children with cerebellar developmental anomalies, in another cognitive domain: music. In their study they not only observe deficits but also correlations between musical perception, singing performance, and oro-bucco-facial motor control. Using a spiking neural network computation model and simulated cerebellar mediated task, Geminiani et al. elucidate potential contributions of cerebellar dysfunction in the movement disorder dystonia. Thus, combined behavioral and modeling studies can help us in the future to better target which signals are important to emphasize or compensate for in therapeutic development.

Mechanistic studies using cellular physiology and molecular biological approaches can give insights into the function and dysfunction of the cerebellum. Bushart and Shakkottai propose a novel hypothesis to explain why genetic mouse models of human neurodegenerative disease often do not recapitulate the phenotypic neurodegeneration and sometimes ataxia that is a hallmark of the human disorder. They posit an intriguing hypothesis that this may arise from differences in the allometric scaling of the channels within cells of the cerebellum between humans and rodents, usually Purkinje cells (PCs). This enlightening review also provides a compendium of phenotypes of so-called “channelopathies” related to ataxia. Delving further into the mechanisms of neurodegeneration at the molecular level, Borgenheimer et al. detail how pathological changes in PCs in the prototypical spinocerebellar ataxia (SCA) type 1 can pathologically spread to other cell types in the cerebellum, including Bergmann glia, velate astrocytes, and oligodendrocytes. Using an advanced single-nuclei RNA sequencing approach to examine a PC specific mouse model of SCA1, they uncovered that several glial genes involved in shaping PC function were found to be dysregulated, thus suggesting that PC specific changes in function may arise from both cell autonomous and indirect effects. These studies highlight the central role abnormalities at the cellular and molecular level have on generating pathological communication between the cerebellum and forebrain/brainstem.

At the heart of the cerebellum's role across this vast set of behavioral domains is its interconnectivity, receiving and distributing neural information from diverse areas of the brain including the amygdala, basal ganglia, and inferior olive. The studies in this Research Topic offer an insight to cerebellar circuit dysfunction at several levels, point to the possibility of innovative therapeutic approaches by cerebellar modulation or musical training, and may help us in the future to better target areas or sites for clinical remediation.

## Author contributions

All authors listed have made a substantial, direct, and intellectual contribution to the work and approved it for publication.

